# Monitoring land use and soil salinity changes in coastal landscape: a case study from Senegal

**DOI:** 10.1007/s10661-021-08958-7

**Published:** 2021-04-10

**Authors:** Sophie Thiam, Grace B. Villamor, Laurice C. Faye, Jean Henri Bienvenue Sène, Badabate Diwediga, Nicholas Kyei-Baffour

**Affiliations:** 1 Department of Civil Engineering, WASCAL Climate Change and Land Use, Kwame Nkrumah University of Science and Technology, Kumasi, Ghana; 2 Institute of Environmental Sciences, Cheikh Anta Diop University of Dakar, BP 5005 Dakar-Fann, Senegal; 3 Department of Ecology and Natural Resource Management, Centre for Development Research (ZEF), University of Bonn, Walter-Flex St. 3, 53113 Bonn, Germany; 4 Scion, New Zealand Forest Research Institute, Ltd, Titokorangi Drive, Private Bag 3020, Rotorua 3046, New Zealand; 5 Institute National of Pedology, Hann Maristes, Dakar, Senegal; 6 Laboratory of Botany and Plant Ecology, University of Lomé, Lomé, Togo; 7 Department of Agricultural Engineering, Kwame Nkrumah University of Science and Technology, Kumasi, Ghana

**Keywords:** Soil salinity change, Land use intensity analysis, Remote sensing indices, Coastal areas, Senegal

## Abstract

Soil salinity is a major issue causing land degradation in coastal areas. In this study, we assessed the land use and soil salinity changes in Djilor district (Senegal) using remote sensing and field data. We performed land use land cover changes for the years 1984, 1994, 2007, and 2017. Electrical conductivity was measured from 300 soil samples collected at the study area; this, together with elevation, distance to river, Normalized Difference Vegetation Index (NDVI), Salinity Index (SI), and Soil-Adjusted Vegetation Index (SAVI), was used to build the salinity model using a multiple regression analysis. Supervised classification and intensity analysis were applied to determine the annual change area and the variation of gains and losses. The results showed that croplands recorded the highest gain (17%) throughout the period 1984–2017, while forest recorded 3%. The fastest annual area of change occurred during the period 1984–1994. The salinity model showed a high potential for mapping saline areas (*R*^2^ = 0.73 and RMSE = 0.68). Regarding salinity change, the slightly saline areas (2 < EC < 4 dS/m) increased by 42% whereas highly saline (EC > 8 dS/m) and moderately saline (4 < EC < 8 dS/m) areas decreased by 23% and 26%, respectively, in 2017. Additionally, the increasing salt content is less dominant in vegetated areas compared with non-vegetated areas. Nonetheless, the highly concentrated salty areas can be restored using salt-resistant plants (e.g., *Eucalyptus* sp*.*,* Tamarix *sp.). This study gives more insights on land use planning and salinity management for improving farmers’ resilience in coastal regions.

## Introduction

Salinity is among the major environmental factors causing land degradation. It is estimated that nearly 831 million hectares of land in the world are affected by salt (Daliakopoulos et al., 2016; Legros, 2009). Soil salinity is one of the common environmental problems that affect agricultural production and land resources mainly in semi-arid and arid areas (Metternicht & Zinck, 2003). Most of the semi-arid and arid areas are located in the sub-Saharan Africa, where low rainfall and high temperature are major factors influencing soil salinity dynamics (Sakadevan & Nguyen, 2010).

Similarly, in Senegal, soil salinity causes soil degradation which subsequently reduces crop yield and affects food security (Fall et al., 2014). In fact, out of the 3.8 million ha of the cultivated lands in the country, 1.7 million ha are affected by salt at the national level (FAO/CSE, 2003) resulting from seawater intrusion. Additionally, land use/land cover changes also a major contributing factor to soil degradation in Senegal. Also, changes in the vegetation cover and extent of the salt marshes have considerably contributed to expansion of salt-affected areas which will result in further environmental degradation (Masoud & Koike, 2006). Furthermore, intensive use of natural resources in areas where local communities depend on land for agricultural purposes and excessive logging or deforestation are practices that increase environmental degradation in the country (Masoud & Koike, 2006). Indeed, soil salinity becomes a land use/land cover (LULC) issue when it inhibits plant growth, causing death of nearby trees and therefore contributes to the changes and transitions among land use types (Allbed et al., 2017). Moreover, soil salinity is also influenced by the adverse effects of climate change (i.e., sea level rise), and thus making it difficult to monitor and mitigate. In view of this, the continuous and long-term monitoring of the land use change is considered as an essential step for understanding soil salinity change and its effects on other land use types.

Application of remote sensing in detecting and monitoring land use and soil salinity changes has been very useful over the years (Allbed & Kumar, 2013). Since 1960, remote sensing has been progressively applied to monitor and delineate salt-affected areas using aerial photos (Saleh, 2017). Currently, a variety of remote-sensing data and sensors have been developed and used to delineate salt-affected areas. Among them are LANDSAT, SPOT, IKONOS, and Terra-ASTER with the resolution ranging from medium to high as well as hyperspectral sensors (Metternicht & Zinck, 2003; Azabdaftari & Sunar, 2016). In recent years, various salinity and vegetation indices such as normalized difference vegetation index (NDVI), normalized difference salinity index (NDSI), salinity index (SI), and soil-adjusted vegetation index (SAVI) have been used to delineate salt-affected areas (Zhang et al., 2011; Taghadosi & Hasanlou, 2017; Yossif, 2017; Allbed et al., 2017). By integrating multi-temporal imagery with biophysical data, these remote-sensing indices and soil properties may provide an accurate estimation of soil salinity changes. In this study, Landsat imagery data were used for the assessment as recommended in many land use change studies (Wu et al., 2008; Narmada et al., 2015; Allbed et al., 2017; Emad & Emad, 2017).

In Senegal, many land use change studies have been carried out using remote-sensing techniques (Sylla, 1994; Wiegand et al., 1996; Parton et al., 2004; Abdul Qados, 2011; Sambou et al., 2016; Faye et al., 2016; Sambou et al., 2016; Barry et al., 2017). The results of these researches showed major changes in land use patterns over the past years. For example, the loss of mangrove and forested areas, expansion of agricultural lands and salt marshes, shift of islands, and loss of traditional rice fields mainly due to inappropriate land management (i.e., illegal tree logging), frequency of flooding and drought, and soil degradation (i.e., salinity and erosion). Despite the changes in land use patterns, the relationship between changes in soil salinity and land use changes remains lacking. Apparently, the Saloum river region, particularly Djilor district, as a coastal area, has suffered from salinization as a result of seawater intrusion, insufficient rainfall, and increased temperature since the drought of 1970 (Sambou et al., 2016). The salt accumulation process, in this zone, has led to the formation of saline soils, which makes the soil unsuitable for agricultural production (Thiam et al., 2019). Hence, complementing land use change assessment with soil salinity analysis may provide important information to identify land management practices that helps to cope with the negative impacts of soil salinity increase. For that purpose, this paper aims at investigating the soil salinity dynamics together with land use changes between 1984 and 2017 in Djilor district. These specific objectives were set out to help achieve the aims: (1) assess the land use/land cover changes and (2) build a soil salinity predictor model for salinity change estimation and mapping using satellite images, biophysical, and soil data.

## Materials and methods

### Study area

**Fig. 1:**
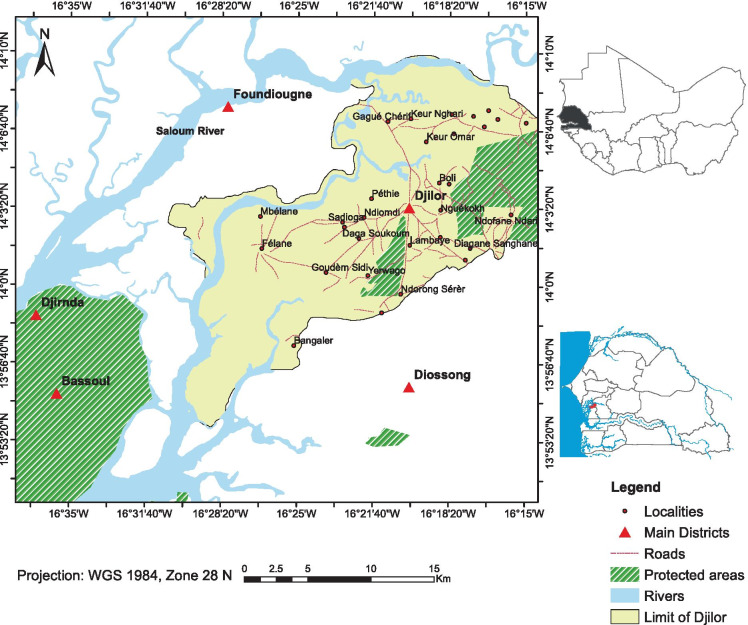
Location of Djilor district, Fatick Region (data source: National Institute of Pedology)

The study was carried out in Djilor district in Fatick Region at the west-central part of Senegal. It is located between latitude 13°54 and 14° 04′ N and longitude 16°12 and 16°20′ W (Fig. 1). Djilor covers a total area of 444 km^2^ and bounded to north west by the Saloum River. It is located about 40 km from the sea and situated within the Saloum Delta Biosphere Reserve, which combines the characteristics of costal, estuarine, and lacustrine landscapes (PLD, 2009). Salinization has been noticed in many parts of the area, mainly due to seawater intrusion from the Saloum River and evaporation that has a great impact on soil fertility and crop production (Faye et al., 2005); this endangers the livelihoods and food security of communities (Sambou, 2016). Groundnuts, millet, maize, sorghum, and rice are the main crops in Djilor. It is located in the Sudan-Sahelian zone, which is characterized by uni-modal rainy season from June to October and a dry season from November to May. Based on historical observations for the period of 1965 to 2016, the annual mean rainfall is estimated to be 546 mm (data source: Senegal National Meteorological Agency). However, the annual rainfall has fluctuated strongly with a major decrease during the period from 1970 to 1990 followed by a slight increase in annual rainfall from 2008 to 2016. In terms of the temperature, the region is characterized by an annual average temperature ranging from 28 to 31 °C. The maximum temperature is noticed in April of about 39.55 °C. During cold periods (January to December), temperature can drop below 20.7 °C (Senegal National Meteorological Agency, 2016).

Djilor is characterized by three dominant soil types: Lixisols (tropical ferruginous soils), Gleysols (hydromorphic soils), and Fluvisols (halomorphic soils). The tropical ferruginous soils, commonly known as “*Dior*” soils in Djilor, cover 52% of the total area, while hydromorphic soils, locally called “*Deck soils*”, cover 9%. The halomorphic soils known as acid sulfate soils also called “*tann*” in Djilor cover 29% (PLD, 2009).

The coastal strands, tidal flats, depressions, and terrace uplands are the main geomorphological units of the area. The tidal channels and the topography mostly dominated by low land in the region; this has facilitated the degradation of the environment and the intrusion of saltwater into agricultural land (PLD, 2009).

### Estimation of land use/land cover change

#### Data source and pre-processing

Four Landsat images for the years 1984, 1994, 2007, and 2017 were downloaded from United States Geological Survey and used to assess the patterns of land use/land cover (LULC). These images were acquired during the dry season between March and April to enable a clear distinction of features, especially salt surface features (Lhissou & Chokmani, 2014). All the pre-processing including geometric and atmospheric corrections (Abd El-Kawy et al., 2011) and processing of the images were made using ERDAS IMAGINE 14. The images were all georeferenced to UTM WGS 1984 projection system. To train and validate the classified maps, a set of 164 points were collected by random sampling to represent the different LULC types using handheld GPS (Faye et al., 2016). A total of 114 points representing 70% were used for training purpose and the remaining 30% for validation (Abdi, 2020). The field work was performed from April to May 2017 to collect information on historical LULC and validate the classified images using visual interpretation with Google earth historical images and local knowledge from key informants in the study area.

#### Data processing and analysis

A supervised classification was performed. The signature and the number of classes for the supervised classification were developed based on the field investigation and the existing LULC classification map collected from the Centre of Environmental Monitoring (Centre de Suivi Ecologique). Eight main categories of land use/land cover types were identified and classified (Table 1). The accuracy assessment of the classification was checked by computing the confusion matrix, the overall accuracy, and the kappa coefficient for each year as well as the errors of omission and commission (Diwediga et al., 2017). To minimize classification errors due to image registration, all the classified maps were subjected to 3 × 3 pixels filtering to have a good homogeneity (Faye et al., 2016). The classified images were exported to ArcGIS for enhancement and mapping of LULC types of each year. Stepwise, a post-classification comparison was used for land use/land cover change (LUCC) detection.

**Table 1:** Description of the LULC types

Value	LULC	Description
1	Mangrove	Mangrove and estuaries with aquatic vegetation dominated by *Rhizophora racemosa, Rhizophora mangle*, and *Avicennia Africana*
2	Savannah/shrubs	Vegetation composed of tree savannahs, shrubs, and grasslands. Generally, tree height is lower than 5 m
3	Forests	Woodland or protected areas with trees height higher than 5 m
4	Salt mashes	Soil salt marshes, corresponding to the tidal areas and generally submerged They are bordered by sabkhas and occur along the coast
5	Sabkha	Local term for tann, which soils are with salt crust on surface salt flat soils, characterized by very poor vegetation cover composed mainly of halophytes
6	Water bodies	Rivers, reservoirs and lagoons
7	Bare lands	Abandoned areas, settlements
8	Croplands	Cereal crops and vegetables crops (e.g., rice, millet, maize, and groundnut)

Once the land use/land cover classification was established, the intensity analysis method was applied (Aldwaik & Pontius, 2012). We particularly applied the interval and category levels to determine the time intervals during which the annual change area is relatively slow versus fast, and the variation of the categories’ gains and losses during a time interval, respectively. Interval and category level analyses were performed using the following equations:

Equations 1 and 2 give the uniform intensity (*U*) across time extent (*Y*_1_, *Y*_T_) and the annual change (*S*_t_) for each time interval (*Y*_t_, *Y*_t+1_), respectively. If *S*_t_ > *U*, then the change is fast for (*Y*_t_, *Y*_t+1_), if *S*_t_ < *U*, then the change is fast for (*Y*_t_, *Y*_t+1_), and if *S*_t_ = *U* for all time interval, then the annual change is stationary. As well, the category level was computed, this determines the variation of the categories’ gains and losses during a time interval.

Equations 3 and 4 were used to calculate the change in terms of loss (*L*_ij_) and gain (*G*_ij_) for the four time intervals (Diwediga et al., 2017; Villamor et al., 2013).

1 $$U=\frac{\left(\mathrm{change}\;\mathrm{area}\;\mathrm{during}\;\mathrm{all}\;\mathrm{inteval}\right)\;100}{\left(\mathrm{duration}\;\mathrm{of}\;\mathrm{all}\;\mathrm{interval}\right)\mathrm{domain}\;\mathrm{area}}$$

$$=\frac{{\sum }_{\mathrm{t}=1}^{\mathrm{T}-1} \left\{{\sum }_{\mathrm{j}=1}^{\mathrm{J}}\left[\left(\sum_{\mathrm{i}=1}^{\mathrm{J}}{C}_{\mathrm{tjj}}\right)- {C}_{\mathrm{tjj}}\right]\right\} 100}{\left({Y}_{\mathrm{T}}-{Y}_{1}\right)\sum_{\mathrm{j}=1}^{\mathrm{J}}{\sum }_{\mathrm{i}=1}^{\mathrm{J}}{C}_{1\mathrm{ij}}}$$

2 $$S_{\mathrm t}=\frac{\left(\mathrm{change}\;\mathrm{area}\;\mathrm{during}\;\left[Y_{\mathrm t},Y_{\mathrm t+1}\right]\right)\;100}{\left(\mathrm{duration}\;\mathrm{of}\;\left[Y_{\mathrm t},Y_{\mathrm t+1}\right]\right)\mathrm{domain}\;\mathrm{area}}$$

$$=\frac{{\sum }_{\mathrm{j}=1}^{\mathrm{J}}\left[\left({\sum }_{\mathrm{i}=1}^{\mathrm{J}}{C}_{\mathrm{tij}}\right)- {C}_{\mathrm{tjj}}\right] 100}{\left({Y}_{\mathrm{t}+1}-{Y}_{\mathrm{t}}\right)\sum_{\mathrm{j}=1}^{\mathrm{J}}{\sum }_{\mathrm{i}=1}^{\mathrm{J}}{C}_{\mathrm{tij}}}$$

where *U* = the uniform annual change during extent [*Y*_t_, *Y*_T_]; *S*_t_ = the annual change during interval [*Y*_t_, *Y*_t +1_]; *T* = number of time points, which equals 4 for this study; *Y*_t_ = year at time point *t*;* t* = index for the initial time point of interval [*Y*_t_, *Y*_t+1_], where *t* ranges from 1 to *T – 1*; *J* = number of categories; *i* = index for a category at an interval’s initial time point; *j* = index for a category at an interval’s final time point; *C*_tij_ = number of pixels that transition from category *i* to category *j* during interval [*Y*_t_, *Y*_t +1_].

3 $$L_{\mathrm{ij}}=\left(P_{\mathrm i}-P_{\mathrm{ii}}\right)\left(\frac{P_{\mathrm j}}{\sum_{\mathrm j=1}P_{\mathrm j}}\right),\mathrm{where}\;i\neq j$$

4 $$G_{\mathrm{ij}}=\left(P_{\mathrm j}-P_{\mathrm{jj}}\right)\left(\frac{P_{\mathrm i}}{\sum_{\mathrm i=1}P_{\mathrm i}}\right),\mathrm{where}\;i\neq j$$

where *L*_ij_ is the proportion of loss from category *i* to *j* under random processes of loss and *P*_ii_ is the proportion of the category *i* that showed persistence between the two times; *G*_ij_ is the proportion of gain from category *i* to *j*, *P*_j_ is the proportion of the landscape in category *j* in the final time; *P*_jj_ is the observed persistent proportion of the category *j*; *P*_i_ is the total area of category *i* at initial time.

#### Estimation of soil salinity change

**Fig. 2:**
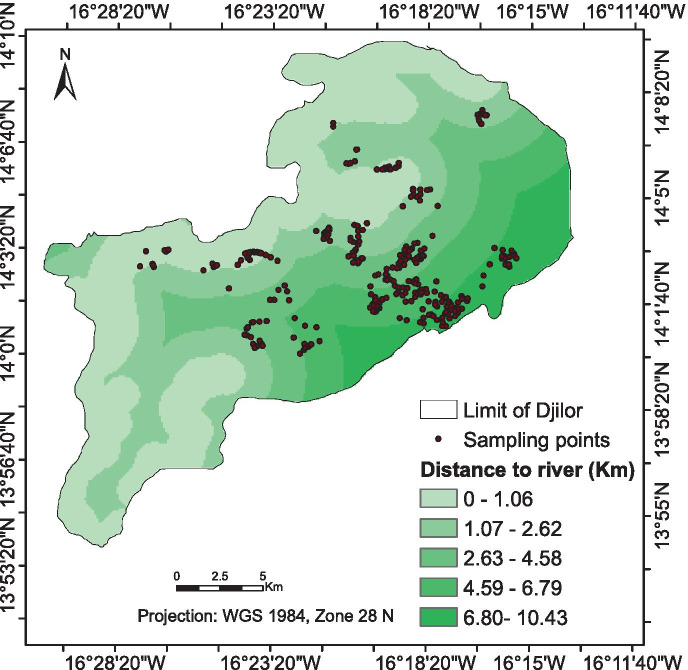
Location of soil sample points

In order to assess the spatial soil salinity change and predict the salinity level at different locations of the study area over the period 1984–2017, a total of 300 composite soil samples were randomly collected from the first top 30 cm (0–30 cm depth) in the different LULC types (Dahal & Routray, 2011) (Fig. 2). These samples were brought to the laboratory to determine the electric conductivity (EC). The soil sample locations were georeferenced, and biophysical characteristics such as elevation, distance to river, wetness index (TWI) as well as remote sensing indices (NDVI), salinity index (SI), and soil-adjusted vegetation index (SAVI) were derived from Landsat image 2017 (Table 2).

**Table 2:** Remote sensing indices

Index name	Formula	Source
Normalized differential vegetation index (NDVI)	$\frac{\mathrm{Band}\;4-\mathrm{Band}\;3}{\mathrm{Band}\;3+\mathrm{Band}\;4}$	Tripathi et al*. *(1997)
Soil-adjusted vegetation index (SAVI)	$\frac{\mathrm{Band}\;4-\mathrm{Band}\;3}{\left(\mathrm{Band}\;3+\mathrm{Band}\;4+L\right)}(1+L)$	Wilson et al. (2016)
Salinity index (SI)	$\sqrt{\mathrm{Band}\;3\times\mathrm{Band}\;4}$	Dehni and Lounis (2012)

These indices were chosen because they have given better correlation in the analysis of salt-affected areas and constitute good indicators for salinity classification and quantification (Poenaru et al., 2015). They were recently used in various regions to predict soil salinity distribution (Zhang et al., 2011; Taghadosi & Hasanlou, 2017; Yossif, 2017; Allbed et al., 2017). Elevation and TWI were derived from DEM (i.e., 30 m × 30 m resolution); DEM was obtained from the Directorate of Geographic and Cartographic Services in Senegal. Distance to the river was generated using Euclidian distance module in ArcGIS 10.3. The spatial analysis tool in ArcGIS was used to extract NDVI, SAVI, and SI values corresponding to each EC sampled point. A stepwise regression analysis was applied where remote sensing indices (SI, SAVI, and NDVI) and biophysical characteristics (elevation, distance to the river, and TWI) are independent variables whereas EC values are the dependent variable. At first, we included all these variables and conducted a stepwise regression in order to find the best fit model. The predicted EC generated from the regression model that showed the best correlation with EC was used to map soil salinity for the years 1984 and 2017. However, the regression salinity model was not applied to the 2007 image in order to reduce uncertainty in the change analysis (Yossif, 2017).

The classification of salinity level is based on the global standard salinity ranges (Azabdaftari & Sunar, 2016). From that, four salinity classes were considered in mapping salinity level (Table 3).

**Table 3:** Categorization standard of soil salinity (Azabdaftari & Sunar, 2016)

Salinity level (dS/m)	Salinity class
>8	Highly saline
4–8	Moderately saline
2–4	Slightly saline
<2	Non-saline

## Results

### Land use/cover change over the period 1984–2017

**Fig. 3:**
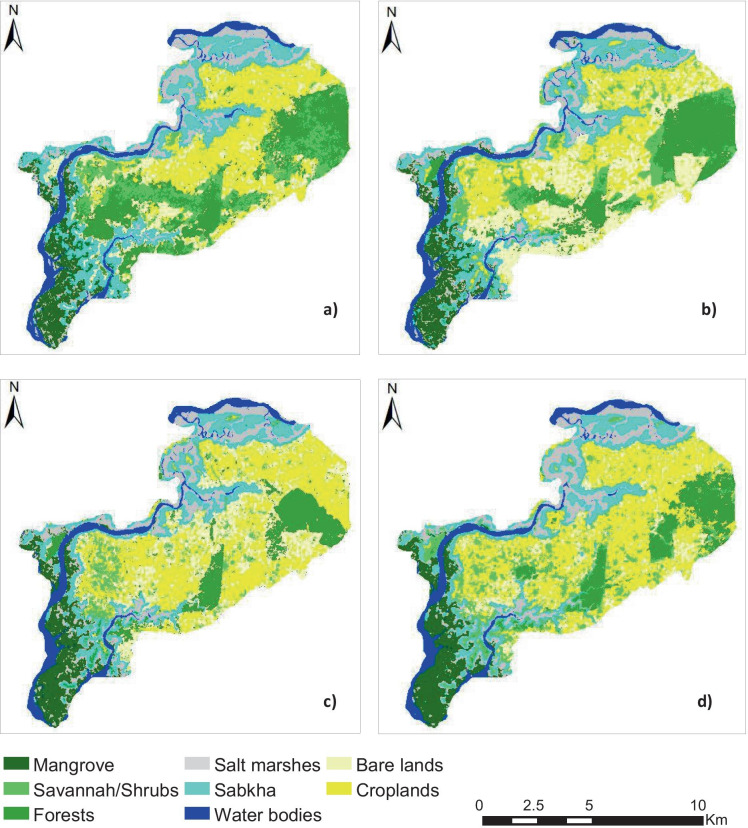
Historical land use/land cover types in Djilor for the year 1984 (**a**), 1994 (**b**), 2007 (**c**), and 2017 (**d**)

Figure 3 represents the land use/land cover maps of 1984, 1994, 2007, and 2017 of Djilor whereas their respective land use/land cover changes are shown in Table 4. Croplands and forests constitute the major land cover types with 20% and 18% of the total area in 1984, respectively. In 2017, cropland experienced the largest gain of 30% of the total area (Table 4).

**Table 4:** Land use/land cover statistic from 1984 to 2017

LULC	1984		1994		2007		2017	
	ha	%	ha	%	ha	%	ha	%
Mangrove	2920	6.87	3158	7.43	3337	7.86	3876	9.13
Savannah/shrubs	3908	9.20	3063	7.21	1836	4.32	3539	8.33
Forests	7755	18.26	5895	13.88	4109	9.67	3881	9.14
Salt marshes	4905	11.55	4829	11.37	5532	13.02	5550	13.07
Sabkha	6008	14.14	4959	11.68	4492	10.58	5128	12.07
Water bodies	2591	6.10	2868	6.75	2941	6.92	2740	6.45
Bare lands	5886	13.86	9902	23.31	9268	21.82	5050	11.89
Croplands	8502	20.02	7801	18.37	10,959	25.80	12,711	29.93
Total	42,475	100	42,475	100	42,474	100	42,475	100

Areas in ha are rounded to avoid decimal figures

The reliability of these different statistics has been confirmed by the accuracy assessment using kappa and the overall accuracy. The classification shows an overall accuracy of 86.58%, 88.13%, 97.22%, and 99.68%, respectively, for the years 1984, 1994, 2007, and 2017 (see Appendix 1). The results of kappa coefficients range between 0.86 and 0.99, and overall accuracy is much enough and satisfactory to confirm the accuracy classified maps. However, the mangroves, salt marshes, and Sabkha were the most accurate classified land use types over time, while forest and savannah registered some classification errors mostly due to the confusion between them.

**Fig. 4:**
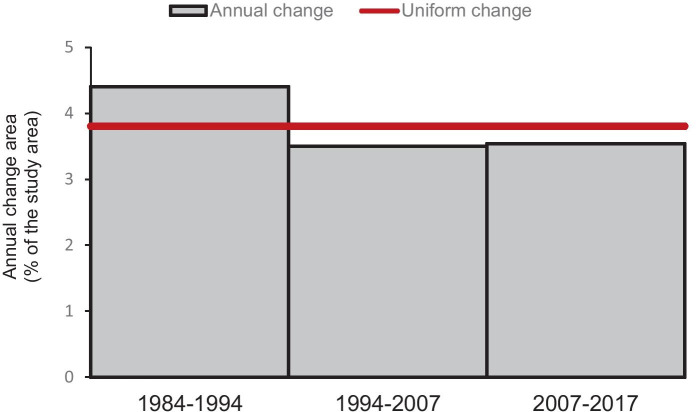
Interval level change intensity as an annual percent of the study

In terms of interval level, Fig. 4 shows that the annual area of change between 1984 and 1994 is faster than the annual area of change between 1994–2007 and 2007–2017. This can be attributed to forest loss and the gains of croplands and bare lands.

**Fig. 5:**
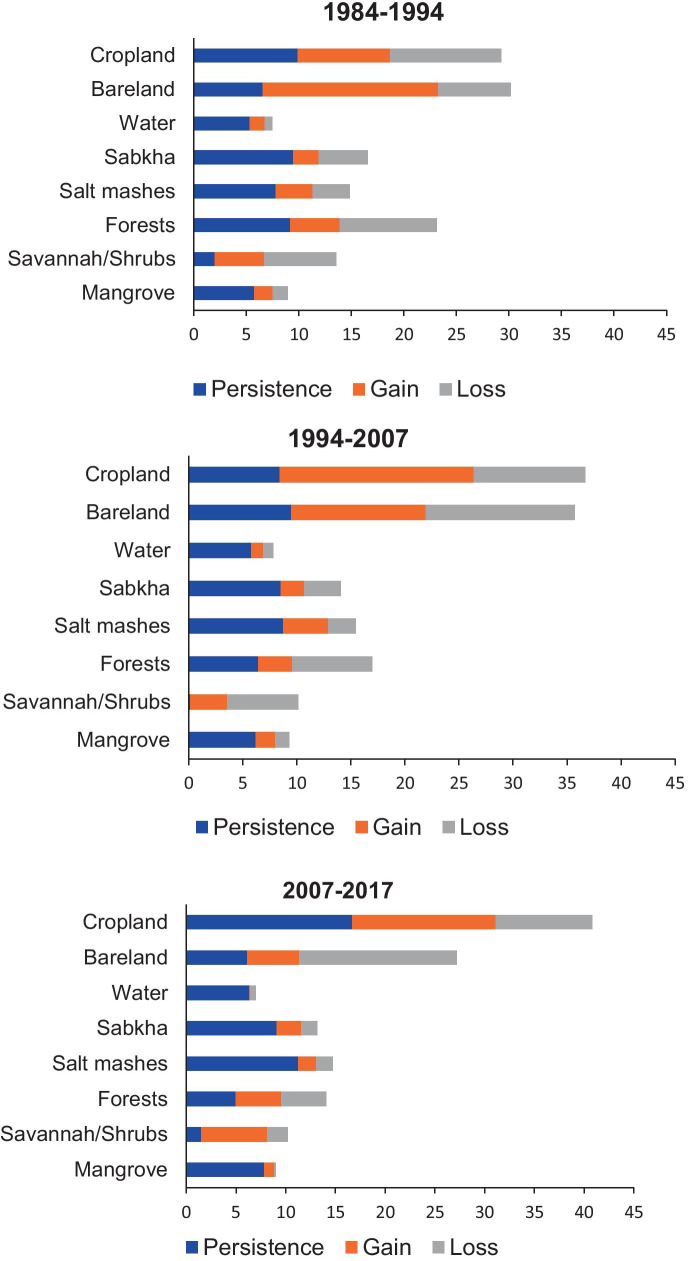
Land use change persistence, gains, and losses for four-time periods

Figure 5 presents the gains, losses, and persistence of the major land use types for three-time intervals. During the first-time interval (1984–1994), bare lands registered the highest gain (16.7%) followed by croplands (8.8%). In contrast, forests and croplands had more losses compared with gains. During the second time interval (1994–2007), croplands and bare lands had both the highest gains and losses suggesting that these land use types were highly dynamic. Within this time interval, forests decreased its cover by 6.4% as compared with the first-time interval (9.2%). During the last time interval (2007–2017), croplands experienced the largest gain (14.5%), followed by savannah (6.6%). Contrarily, bare lands (15.8%) had the highest loss followed by croplands (9.7%).

### Soil salinity predictor model

**Fig. 6:**
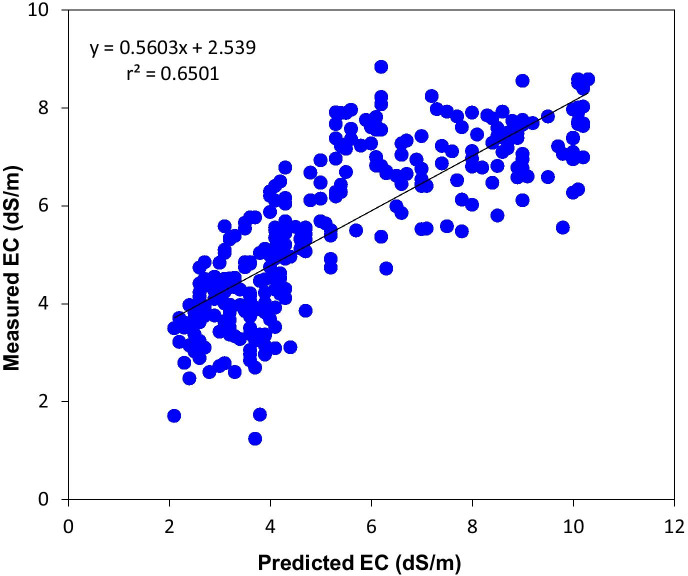
Correlation between measured EC and predicted EC values

The selected regression model (Eq. 5) combining biophysical data and NDVI gives a greater correlation with EC (*R*^2^ = 0.73) compared with SI (*R*^2^ = 0.57) and SAVI (*R*^2^ = 0.55) (see Appendix 2). The distance to the river, elevation, and NDVI was significantly associated with EC (*P* < 0.05). The statistical significance of the regression is at the 0.05 level. The regression analysis has significant estimation (Prob > *F* = 0.000) and good fit in the area with *R*^2^ = 0.73 and RMSE = 0. 68. The test for validation of the regression model showed a strong correlation between measured EC and predicted EC values, with the coefficient of correlation (*r*^2^ = 0.65) (Fig. 6). Hence, the regression model (Eq. 5) can be used to predict and map the salt-affected areas in the study area.

5 $$\mathrm{EC}=9.98-0.0005\times d-0.20\times \mathrm{elevation}-6.58\times \mathrm{NDVI}+0.00007\times \mathrm{TWI}$$

### Soil salinity change over the period 1987–2017

**Fig. 7:**
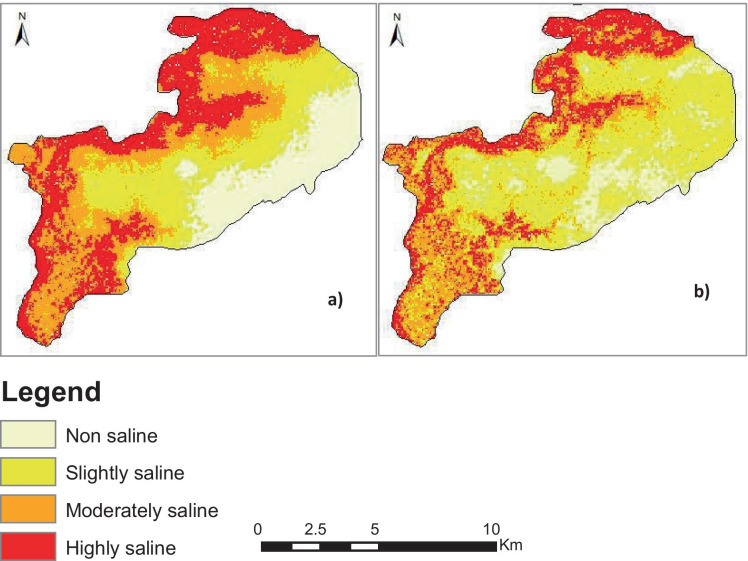
Soil salinity maps for the years 1984 (**a**) and 2017 (**b**)

**Fig. 8:**
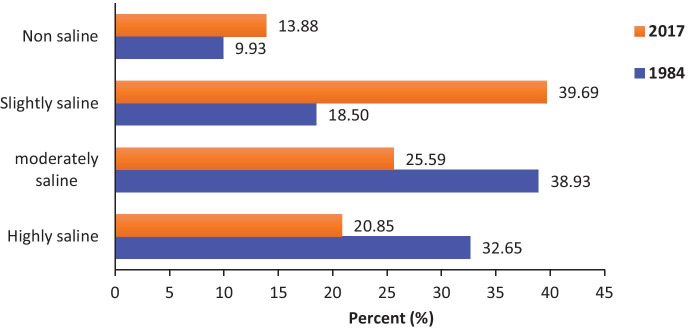
Soil salinity change for the years 1984 and 2017

Figure 7 compares the soil salinity maps for the years 1984 and 2017 with four salinity classes (non-saline, slightly saline, moderately saline, and highly saline). Figure 8 shows the statistics of soil salinity level noticed in the area from 1984 to 2017. In 1984, moderate saline soils registered the higher coverage (165.8 km^2^), corresponding to 38.9% of the area. Highly saline areas cover 32.65% of the total area whereas slightly saline and non-saline soils were lower in coverage representing 18.5 and 9.93%, respectively. In 2017, the slightly saline areas are the highly represented in the area with 39.69%, followed by moderately saline (25.6%). Highly saline and non-saline soils registered, respectively, 20.85 and 13.88%. The period 1984–2017 is characterized by a decrease in salinity level over the area. In fact, slightly saline and non-saline soils gained 42.14 and 7.85%, respectively, between 1984 and 2017, while highly saline and moderately saline areas decreased in their area (−23.47% and −26.53%), respectively.

**Fig. 9:**
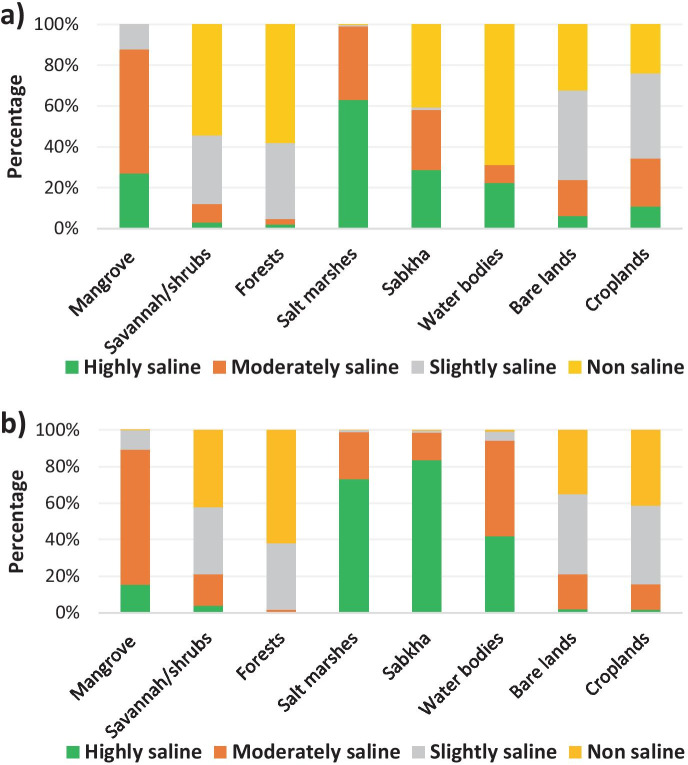
Percentage of salt-affected areas per land use/land cover type for the years 1984 (**a**) and 2017 (**b**)

Figure 9 shows the percentage of salt effected areas per land use types for the years 1984 and 2017. In general, non-vegetated areas (e.g., sabkha, salt marshes, bare lands, and croplands) have a higher coverage of salt-affected areas compared with vegetated areas (e.g., forest, savannah, and mangrove). In 1984, 31.04% and 27.88% of highly saline category areas were contained in sabkha and salt marshes, respectively. Croplands registered 19.13% of the moderately saline category soils, while savannah and forest had the lowest saline areas. In 2017, salt marshes and sabkha registered, respectively, 44.42% and 25.76% of highly saline category, while non-saline areas were more dominant in croplands (45.30%) and forests (24.11%).

## Discussion

### LULC mapping and accuracy

The land use land cover change analysis showed an increase in agricultural (17%) and bare lands (9%) at the expense of forest which was characterized by high loss (12%) over the period 1984–2017. These results reveal the ongoing loss of vegetation in the area as shown by the decreasing trend of forest. Similar results were observed in the neighboring area of Fatick in Senegal (Sambou et al., 2016) and in South-Eastern part of Senegal (Faye et al., 2016). These changes in LULC are mainly due to human activities such as continuous expansion of farms to sustain food production. Also, salinization is a severe phenomenon that has contributed to LUCC and has caused the toxic effects on plant growth (Allbed et al., 2017). This could be seen in the percentage of salt-affected areas per land use/land cover type over the period 1984–2017. Indeed, the sabkha and salt marsh areas registered the largest highly saline coverage with, respectively, 31.04% and 27.88% in 1984 compared with forests (1.50%) and croplands (8.92%). Another important finding of this study was the reduction of highly saline areas by 5.28% and 7.17%, respectively, in sabkha and croplands in 2017. This decrease can be explained by the slight improvement of rainfall in the area since the drought period of 1971, which contributed to leach out the salt from the soils. As well, LULC change was more intense (4.40%) in the first period 1984–1994 (see Fig. 4). Historical evidence may explain this finding, since that period was characterized by an increasing resource pressures and land degradation due to severe events such as drought (Sadio & van Mensvoort, 1993; Faye et al., 2020).

### Soil salinity dynamics

Soil salinity was accurately mapped using multiple regression (Eq. 5) as earlier reported by Morshed et al. (2016). The results show that salinity level has been characterized by a relative decrease between 1984 and 2017 in the region. Indeed, highly saline and moderately saline areas have decreased by 23.47% and 26.53%, respectively. These results confirm the reduction of the extent of salt-affected areas registered in the different land use type in 2017. Such decrease of salinity could be related to the improvement of rainfall recorded in the area (Descroix et al., 2020) as well as the various adaptation and mitigation measures (e.g., anti-salt dams, revegetation and conservation of trees, use of manure and mulching, etc.) implemented by the local communities and some NGOs. Similarly, Sambou et al. (2015) showed slight restoration of affected areas by the construction of anti-salt micro-dam and the improvement in rainfall in Casamance (Southern Senegal). In addition, it was also noticed that year 1984 registered the higher salt-affected areas that may be explained by the deficit in rainfall during that year. In fact, from 1971 to 1985, the Sahel in general, particularly Senegal, has been through a severe drought period characterized by a drastic reduction in rainfall which have contributed to the expansion of salt-affected areas in the country (Sadio & van Mensvoort, 1993). Our finding is in accordance with the observation of Kairis et al. (2013) which reported that areas with low amounts of rainfall (>650 mm) are more likely to be affected by salinity. This indicates that the decrease in rainfall resulting from climate change could accelerate salinization, but this requires further investigation in the study area. This indicates that, as a result of climate change, salinization may be hindered due to decreased rainfall and further research is required.

Additionally, the spatial distribution of salinity in Djilor showed that the highly saline areas are mostly located along the river, generally corresponding to the salt marshes and sabkha areas. Suggesting that the salinity gradient is mostly horizontal and gradually moving from the river to the uplands (Manandhar & Odeh, 2014). These findings support the fact that soil accumulation in this region is generally caused by inundation and deposits of salt from seawater intrusion combined with a high temperature. Also, our results show that vegetation cover is a determining factor of spatial distribution of salinity in the area (Ivushkin et al., 2019). In fact, patches of non-saline soils are more pronounced in vegetated areas (Forests and Savannah), compared with non-vegetated areas (bare land and sabkha) which registered high content of salt. This finding corroborates with a study in Saudi Arabia (Oasis), which reported that vegetated areas exhibited the lower salinity, while high salinity was exhibited in non-vegetated areas (Allbed et al., 2017, 2014). This may suggest that planting salt-tolerant tree species may further reduce salinity but further need investigation.

Among the remote-sensing indices, NDVI appears relevant in the assessment of salinization because it gave a higher correlation (*R*^2^ = 0.73) compared with SI (*R*^2^ = 0.57) and SAVI (*R*^2^ = 0.55). These results are similar to those of Emad and Emad (2017) in northern Egypt and Jabbar and Zhou (2012) in Southern Iraq, who reported that NDVI index gives good results with assessing soil salinity. However, we have noticed during the stepwise regression that NDVI, coupled with biophysical data such as elevation, and distance to the river give high *r*-squared (*R*^2^). This result confirms the strong influence of biophysical factors in the expansion of salt-affected areas in Djilor and their importance in monitoring soil salinity in coastal regions (Thiam et al., 2019).

## Conclusion

In this paper, we investigated the spatial patterns and dynamics of LULC, in salt-affected areas, over the period 1984–2017. Our results show that the dynamics of LULC in Djilor is characterized by an increase in croplands (17%) at the expense of forest cover (12% of loss).

These changes attest the ongoing deforestation in the area and the continuous expansion of farms by local communities who promote to soil degradation by expanding agricultural lands to sustain food production. Annual area of land cover change is faster during period 1984–1994 (4.40%) than during 1994–2007 (3.50%) and 2007–2017 (3.52%). Historical evidence explains this finding, since there were increasing resource pressures and land degradation in 1984–1994 due to severe events such as drought.

Furthermore, changes in soil salinity level for the years 1984 and 2017 revealed a slight decrease. The highly saline areas decreased by 23.47% while slightly saline and non-saline areas gained 42.14% and 7.85%, respectively. However, despite this decrease, soil salinity remains one of the main factor of soil degradation in the study area as salt-affected areas (i.e., highly saline and moderately saline areas) cover around 60% and 45% of the total area of Djilor in 1984 and 2017, respectively. Spatial distribution of soil salinity is mostly related to vegetation in the area. In fact, the highly saline soils were mostly located in the non-vegetated areas (Sabkha, Salt marshes, Croplands) while non-saline areas are situated in the vegetated areas (Forests and Savannah).

This paper gives a clear understanding of land use/land cover and soil salinity dynamics in Djilor, resulting from land management. Indeed, the results show a high potential of integrating remote sensing and field data to assess soil salinity. The findings are useful for guiding decision makers, land planners, and smallholder farmers to reverse vegetation decline and restore salt-affected areas through the adoption of land management practices as well as integrating new strategies for improving people’s livelihood. Since vegetation is playing an important role in salinity distribution, more efforts should be done on regeneration of salt resistant tree species (e.g., *Eucalyptus *sp.,* Acacia *sp.,* Tamarix *sp.). With regards to the future effects of climate change (increased in temperature and sea level rise and decrease in rainfall), further investigations on modeling future soil salinity as well as assessing the impacts of some adaptation strategies on soil salinity may be useful for sustainable soil salinity management in the study area.

## Appendix 1 Accuracy assessment of the classified LULC maps (1984, 1994, 2007, and 2017)

**Table:**

Land use/cover types		Ground truth (pixels)								Accuracy assessment			
		Mangroves	Savannah	Forests	Salt marshes	Sabkha	Water bodies	Bare lands	Croplands	Prod Acc. (%)	Users Acc. (%)	Ov Acc (%)	Kappa
1984 classified data													
	Mangroves	123	0	0	0	0	0	0	0	100	100	86.58	0.85
	Savannah	0	102	110	0	0	0	0	0	73.91	48.11		
	Forests	0	18	196	0	0	0	0	0	64.05	88.29		
	Salt marshes	0	0	0	152	0	0	0	0	86.36	100		
	Sabkha	0	0	0	24	128	0	0	0	100	84.21		
	Water bodies	0	0	0	0	0	163	0	0	100	100		
	Bare lands	0	18	0	0	0	0	107	0	100	85.60		
	Croplands	0	0	0	0	0	0	0	177	95.68	100		
1994 classified data													
	Mangroves	124	0	0	0	0	0	0	0	100	100	88.13	0.86
	Savannah	0	32	84	0	0	0	0	40	30.48	20.51		
	Forests	0	0	265	0	0	0	0	0	75.50	97.79		
	Salt marshes	0	0	0	133	0	0	0	0	100	100		
	Sabkha	0	0	0	0	217	0	0	0	100	100		
	Water bodies	0	0	0	0	0	372	0	0	100	100		
	Bare lands	0	1	0	0	0	0	132	2	100	96.35		
	Croplands	0	66	0	0	0	0	0	218	83.85	76.76		
2007 classified data													
	Mangroves	105	0	0	0	0	0	0	0	100	100	97.22	0.97
	Savannah	0	122	0	0	0	0	0	1	100	99.19		
	Forests	0	0	337	0	0	0	12	0	94.40	96.56		
	Salt marshes	0	0	0	119	0	0	0	0	100	100		
	Sabkha	0	0	0	0	150	0	0	0	95.54	100		
	Water bodies	0	0	0	0	0	271	0	0	100	100		
	Bare lands	0	0	17	0	2	0	130	0	91.55	87.25		
	Croplands	0	0	3	0	5	0	0	165	99.40	95.40		
2017 classified data													
	Mangroves	136	0	0	0	0	0	0	0	100	100	99.68	0.99
	Savannah	0	158	0	0	0	0	0	0	100	100		
	Forests	0	0	174	0	0	0	0	0	100	100		
	Salt marshes	0	0	0	179	0	0	0	0	100	100		
	Sabkha	0	0	0	0	139	0	0	0	100	100		
	Water bodies	0	0	0	0	0	173	0	0	100	100		
	Bare lands	0	0	0	0	0	0	136	3	99.27	97.84		
	Croplands	0	0	0	0	0	0	1	174	98.31	99.43		

## Appendix 2 Soil salinity predictors from the regression analysis

**Table:**

*R*^2^ of models	Explanatory variables	Coef	Std. Err	95% Conf. interval	
*R*^2^ = 0.73	Intercept	9.980	0.633***	8.735	11.225
	Distance to river (m)	−0.001	0.000***	−0.001	0.000
	Elevation (m)	−0.209	0.040***	−0.287	−0.131
	NDVI	−6.582	0.023***	−14.499	1.335
	TWI	0.000	0.000	0.000	0.000
*R*^2^ = 0.57	Intercept	5.646	3.757***	−1.748	13.042
	Distance to river (m)	−0.000	0.000***	−0.000	−0.000
	Elevation (m)	−0.186	0.037***	−0.262	−0.113
	SI	0.000	0.000	−0.000	0.000
	TWI	0.000	0.000	−0.000	0.000
*R*^2^ = 0.55	Intercept	7.267	2.616***	2.117	12.416
	Distance to river (m)	−0.000	0.000***	−0.000	−0.000
	Elevation (m)	−0.187	0.037***	−0.261	−0.112
	SAVI	6.622	0.000	−0.000	0.000
	TWI	0.000	0.000	−0.000	0.000
